# Comparative oncology chemosensitivity assay for personalized medicine using low-coherence digital holography of dynamic light scattering from cancer biopsies

**DOI:** 10.1038/s41598-024-52404-w

**Published:** 2024-02-08

**Authors:** Zhen Hua, Zhe Li, Dawith Lim, Ali Ajrouch, Ahmad Karkash, Shadia Jalal, Michael Childress, John Turek, David Nolte

**Affiliations:** 1 Department of Physics, Purdue University, West Lafayette, USA; 2 Division of Hematology/Oncology, Indiana University School of Medicine, Indianapolis, USA; 3 Department of Veterinary Clinical Sciences, Purdue University, West Lafayette, USA; 4 Department of Basic Medical Sciences, Purdue University, West Lafayette, USA

**Keywords:** Biophysics, Interference microscopy

## Abstract

Nearly half of cancer patients who receive standard-of-care treatments fail to respond to their first-line chemotherapy, demonstrating the pressing need for improved methods to select personalized cancer therapies. Low-coherence digital holography has the potential to fill this need by performing dynamic contrast OCT on living cancer biopsies treated ex vivo with anti-cancer therapeutics. Fluctuation spectroscopy of dynamic light scattering under conditions of holographic phase stability captures ultra-low Doppler frequency shifts down to 10 mHz caused by light scattering from intracellular motions. In the comparative preclinical/clinical trials presented here, a two-species (human and canine) and two-cancer (esophageal carcinoma and B-cell lymphoma) analysis of spectral phenotypes identifies a set of drug response characteristics that span species and cancer type. Spatial heterogeneity across a centimeter-scale patient biopsy sample is assessed by measuring multiple millimeter-scale sub-samples. Improved predictive performance is achieved for chemoresistance profiling by identifying red-shifted sub-samples that may indicate impaired metabolism and removing them from the prediction analysis. These results show potential for using biodynamic imaging for personalized selection of cancer therapy.

## Introduction

The selection of cancer therapy is an intensely personal event experienced *by* the cancer patient that currently is performed largely *without* personalization *for* the cancer patient. The selection of “standard-of-care” therapies by practicing oncologists in the US is virtually uniform across a disease state, yet approximately half of cancer patients (averaged across disease types and stages) do not respond to first-line cytotoxic chemotherapy^1^. Tumor heterogeneity has been one of the main obstacles to the long-standing search for a method to assess the susceptibility of a patient prior to the start of treatment^2,3^. Genetic heterogeneity, which arises during clonal outgrowth, and may be missed by genetic profiling, renders subpopulations of cells within a tumor resistant to treatment. Spatial heterogeneity of the tumor microenvironment, with varying stromal and extracellular matrix content, can modify cell signaling and affect the chemosensitivity of local groups of cancer cells. Phenotypic profiling has emerged as an alternative that can assess systemic response to treatment^4,5^, for instance using biodynamic imaging^6–8^, but spatial heterogeneity of biopsy material remains a challenge. Spatially-resolved biodynamic imaging as performed in Ref.^9^ can identify heterogeneity within millimeter-scale sub-sections of a tumor biopsy, but it misses centimeter-scale spatial heterogeneity across a full biopsy. To address the role of spatial heterogeneity in the biodynamic profiles of cancer patients, this paper presents the analysis of biodynamic phenotypic profiles that were generated for independent spatial sub-samplings of patient biopsies from two different cancers (B-cell lymphoma and esophageal cancer) across two different species (canine and human).

Biodynamic imaging is a form of en face (or full-field) optical coherence tomography (OCT)^10^ performed using off-axis digital holography as the coherence gate^11,12^. The broad illumination of the samples encourages the formation of high-contrast speckle. From living samples, the speckle is highly dynamic, incorporating the different frequencies among a wide range of Doppler frequency shifts caused by intracellular and cellular motion in living tissue^13^. The fluctuation analysis of the dynamic speckle forms the basis of dynamic contrast OCT that has been used to measure subcellular metabolic activity^14–18^ and extracellular matrix remodeling^19^ as well as to monitor the response of living tissues to drugs^20–24^. The frequency range of the Doppler spectrum spans from 10 mHz to 10 Hz corresponding to membrane rearrangements moving at speeds of nanometers per second, up to fast organelles and vesicle transport at speeds of microns per second, respectively. The ultra-low-frequency Doppler spectroscopy, detecting frequency shifts down to one part in 10^16^, requires the phase-sensitive stability provided by digital holography.

The light-scattering spectrum of living tissue appears similar to the diffusive spectrum of Brownian motion obtained in conventional dynamic light scattering, but the physical origins are different. Brownian motion is a Wiener process characterized by mean-square displacements with no associated velocities, whereas intracellular motions have characteristic short-term speeds and persistence lengths longer than a reduced wavelength inside the tissue (typically λ/4πn = 50 nm) that places living tissue in the Doppler light-scattering regime^13^. The motion in living tissue is a random walk actively driven by energetic molecules such as ATP and GTP, creating a diffusive spectrum that is far out of equilibrium and characterized by an effective diffusion constant given by D_eff_ = vτ^2^, where v is the mean-squared speed of the dominant light-scattering component, and τ is the average persistence time for directed motion. When drugs or other treatments are applied to living samples (ex vivo), the motions inside the living tissue are modified, producing changes in the Doppler spectra. These changes can be specific to different types of biological processes, producing signatures (or spectral fingerprints) of the tissue responding to the treatment^25^.

One of the goals of dynamic contrast OCT and biodynamic imaging using digital holography is to identify patients who will not respond to their cancer treatments so that they can be steered away from ineffective and towards beneficial treatments. Living tissue culture or patient biopsies have been used to assess metastatic potential and target expression by applying redox imaging^26–28^ and chemoresistance status by using fluorescence lifetime imaging^29,30^. Correlation of biodynamic signatures to clinical outcomes has been observed independently in canine B-cell lymphoma^8^, human epithelial ovarian cancer^7^, and human bladder cancer^6^, but no comparative study had been undertaken to explore whether there are common spectral signatures across species or across cancer histotypes. In this work, we present the first dynamic holographic profiles performed for human esophageal cancer patients, comparing them with canine B-cell lymphoma results obtained previously. A key result of this work is the identification of spatially heterogeneous phenotypic subsets of patient biopsies that are common to each disease and species, and the use of spatially variable intratumor phenotypes to improve likelihood ratios for predicting how patients will respond to their prescribed chemotherapy before their chemotherapy even begins.

## Results

This study conducted an analysis of a two-species, two-disease trial that analyzed living tissue biopsies from a historical 19-patient preclinical trial of doxorubicin-based chemotherapy for canine B-cell lymphoma^8^ compared with recently-acquired biopsies from a new 28-patient clinical pilot trial of platinum-based chemotherapy for human esophageal cancer. The importance of comparative oncology in current cancer research is based on two aspects of animal disease relative to human: (1) canine cancers are extremely common, with approximately 50% of all dogs older than 10 years dying from cancer^31^; and (2) many canine cancers are homologous to human cancers in their clinical presentation and molecular pathogenesis. Although the selection in this paper of two unrelated diseases of markedly different histogenesis (lymphoma compared to esophageal cancer) was governed by the availability of patient cohorts to our collaboration, the emergence of common phenotypic signatures across these two trials could represent a commonality of the biodynamic Doppler signatures across disease and across species.

The canine preclinical trial enrolled dogs who presented with diffuse large B-cell lymphoma (c-BCL). All dogs were treated with a 25-week chemotherapy protocol of Cyclophosphamide, Hydroxydaunorubicin (doxorubicin), Oncovin (vincristine), and Prednisolone (known as CHOP). The therapy is administered as a combination therapy in humans, but as a sequence of monotherapies in dogs. Progression-free survival (PFS) time was selected as the primary clinical outcome. The human clinical pilot trial enrolled patients who presented with esophageal adenocarcinoma (h-ESO) at the Indiana University School of Medicine (IUSM) Hospital. A pinch biopsy was performed for normal diagnostic purposes of which a small volume was placed in cold growth medium. Within at least 4 h of the surgery, samples were transported in chilled medium from IUSM in Indianapolis to Purdue University. Each patient received carboplatin + paclitaxel (CT) or cisplatin + 5-fluoruracil (CF) combination therapies and all received radiation therapy.

The biopsy sample volumes were approximately 1 cm^3^ for lymphoma and between 30 and 70 mm^3^ cm^3^ for esophageal with a volume variability patient-to-patient of 50%. These were cut into approximately 1 mm^3^ sections to yield up to 32 sub-samples that were immobilized individually in the wells of a 96-well plate using poly-L lysine. Maintaining the metabolic health of the samples for up to 24 h post-surgery is a key protocol that was developed and validated previously for this research^7,32^. After immobilization, the samples are provided fresh buffered growth media and maintained at physiological temperature. Biodynamic measurements were performed on the millimeter-scale sub-samples of the biopsy immobilized in individual wells by applying combination therapies and mono-therapies. Approximately 5 replicate wells per treatment per patient were acquired, including six well that received only growth medium (negative controls). The sub-samples in the wells were monitored for 4 h prior to applying treatment in vitro to establish the baseline spectrum and to monitor sample drift.

Average intracellular motions are extracted through coherence-gated dynamic light scattering using digital holography to acquire stacks of 2000 electronic holograms at a rate of 25 frames per second with an exposure time of 50 ms for a total measurement time of 4 min. Numerical reconstruction of the holograms yields optical sections that are localized by coherence-gating to the center of the millimeter-scale sub-samples (typically 0.5 mm deep) with a longitudinal resolution of approximately 20 microns (the coherence length of the low-coherence light source broadened by roughly a factor of 2 by multiple light scattering). The digitally reconstructed images display fully developed speckle that changes dynamically across the stack of 2000 frames. Each pixel in the image generates a 2000 element time series, and the fluctuating intensities are transformed into fluctuation power spectra spanning from 10 mHz to 12.50 Hz (Nyquist sampling frequency) that are summed in quadrature over the image pixels of a sub-sample in each well. Subsequent wells are measured in sequence, repeated every 40 min for up to 16 h, including 4 h pre-treatment to establish the base spectrum. This creates a data structure for each well that is a power spectrum at each 40-min time loop. A drug-response spectrogram for each well is then generated by taking the difference in log spectral density using an average over the pre-treatment $\overline{S}\left( {\omega ,t_{0} } \right)$ as the baseline, where

1 $$D\left( {\omega ,t} \right) = \log S\left( {\omega ,t} \right) - \log \overline{S}\left( {\omega ,t_{0} } \right)$$

is the differential drug-response spectrogram. For small relative changes, this is equal to the fractional change in spectral content, and most wells show drug-induced spectral changes within about 30%.

Each well-based spectrogram describes a phenotypic response for each millimeter-scale biopsy sub-sample. Each spectrogram for each well was converted to a 40-element feature vector (see “Methods” and Supplementary Information Fig. S3) that captured the spectral changes induced by the drugs as well as overall health metrics for each sample based on their pre-treatment spectra. There is strong covariance among the 40 features, thus they are combined into 12 pooled features based on correlations (see Supplemental Information Fig. S4). The resulting 12-element feature vectors capture the spectral changes across the time–frequency representation of the drug-response spectrograms and become the front-end of all downstream data analysis.

**Figure 1:**
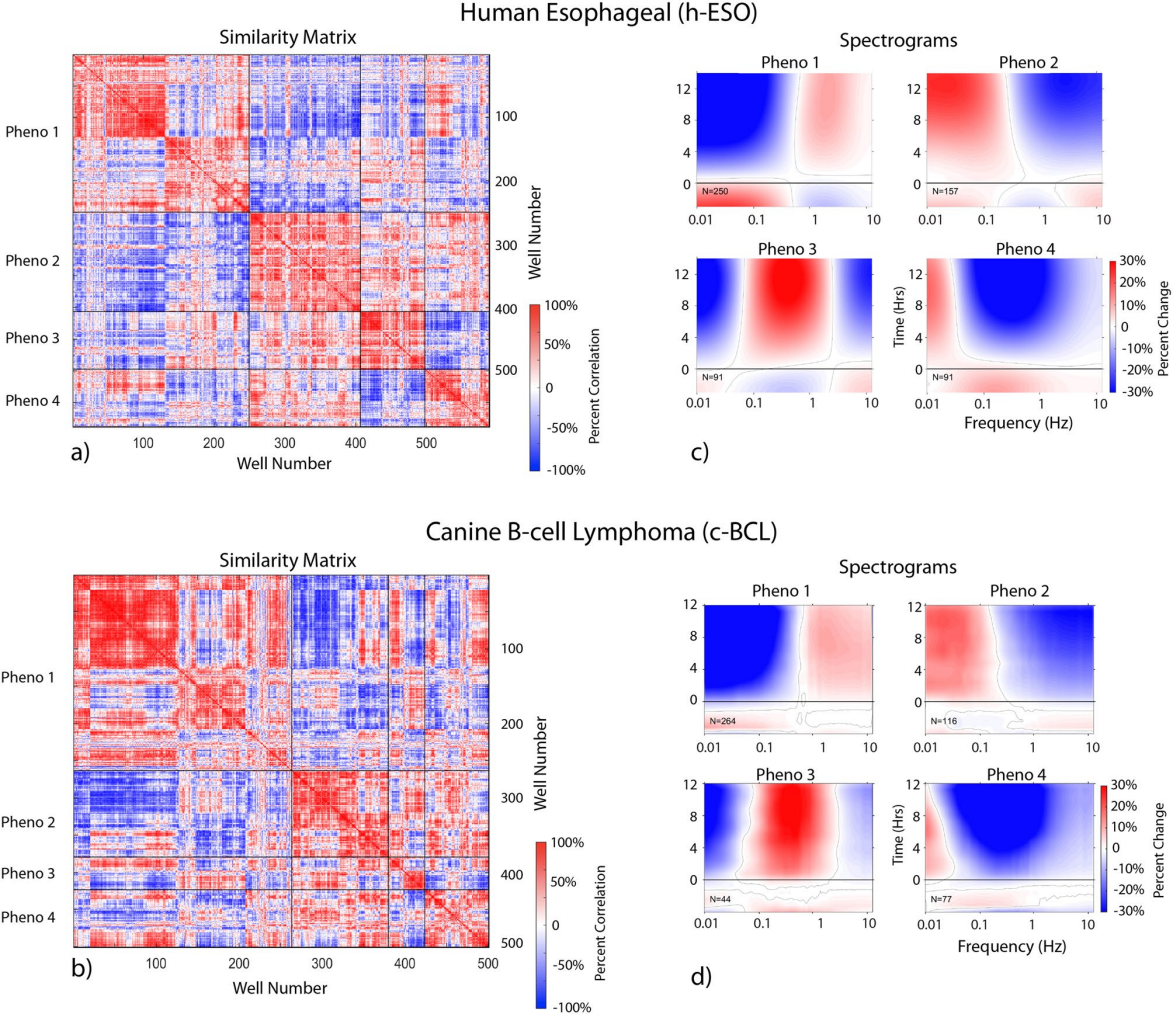
Well-based drug-responses for the human esophageal cancer and canine lymphoma clinical trial. Similarity matrix (**a**) and (**b**) calculated as the correlation between well-based feature vectors from all wells after unsupervised hierarchical clustering by phenotype. Averaged spectrograms D(ω,t) in (**c**) and (**d**) of the four phenotypes. In this nomenclature, phenotype-1 is blue shifted, phenotype-2 is redshifted, phenotype-3 is mid-frequency enhanced, and phenotype-4 is mid-frequency suppressed.

The first step in the data flow is the formation of clustered similarity matrices that describe the similarities among the different wells of a trial. The similarity measure used for this analysis was normalized correlation between the 12-element feature vectors of individual wells. The wells were then clustered using unsupervised hierarchical clustering. The results in Fig. 1a,b show the similarity matrices across the wells. After clustering, the similarity matrices show a roughly block-diagonal structure. The number of clusters is indeterminant, but by pruning the branches of the hierarchical dendrogram into subsets, approximately four main groups emerge for both the canine lymphoma trial and the human esophageal trial. These dendogram sections are delineated by the dark lines on (a) and (b) and are named Pheno 1–4. This clustering of the heterogeneous responses becomes important when one or more of these general phenotypes can be identified with non-characteristic response of the patient to treatment, and this information can be used in the subsequent analysis to improve prediction of patient response to a prescribed chemotherapy.

The average spectrograms of the four phenotypic groups delineated in (a) and (b) are shown in Fig. 1c,d. From a mechanistic perspective, these phenotypes represent the lowest-order Legendre expansions for the functional frequency dependence: Pheno 1 blue shifted (increased organelle activity), Pheno 2 red shifted (decreased metabolic activity), Pheno 3 mid-frequency enhanced (increased membrane activity) and Pheno 4 mid-frequency suppressed (often associated with apoptosis^25^). While it can be argued that there are further subgroups, these same four general phenotypes are observed across the entire set of well-based spectrograms irrespective of the patient cohort (resistant versus sensitive), irrespective of the patient species (human versus canine), and irrespective of the disease type (carcinoma versus lymphoma). Therefore, these four general phenotypes transcend species and cancer type, *representing a potential invariant time–frequency decomposition in biodynamic measurements*. The importance of this result for the general use of biodynamic imaging in chemosensitivity testing, and their likely common origin, is expanded upon in the Discussion.

In the analysis of well-based phenotypes, a phenotype label of 1-through-4 was assigned for each well. A subsequent analysis tested how the phenotype label contributed to predictions of patient chemosensitivity for the resistant-versus-sensitive cohorts. In this comparison, first all wells (all phenotypes) were used to predict patient chemosensitivity, then the analysis was run again by removing wells of one of two sub-phenotypes, either phenotype-2 (red-shift) or phenotype-4 (mid-frequency suppression). The minority sub-phenotype for both trials, phenotype-3 (mid-frequency enhanced), consisted of too few wells to significantly affect the results, while the majority sub-phenotype for both trials, phenotype-1 (blue-shift), consisted of too many wells to censor in the analysis. To predict patient response to therapy, a binary classifier was constructed using a minimal shallow feed-forward neural network (see Supplemental Information^33^) with two layers of 8 hidden neurons that was trained against the clinical outcomes and validated using a 2-holdout cross-validation in which the neural net was trained on all but two patients, and then the two held-out patients were predicted by the neural net and compared to their known clinical outcome. On each run of the analysis, the two hold-out patients were selected randomly, and the analysis was run 60 times, collating the predictions for all the patients.

**Figure 2:**
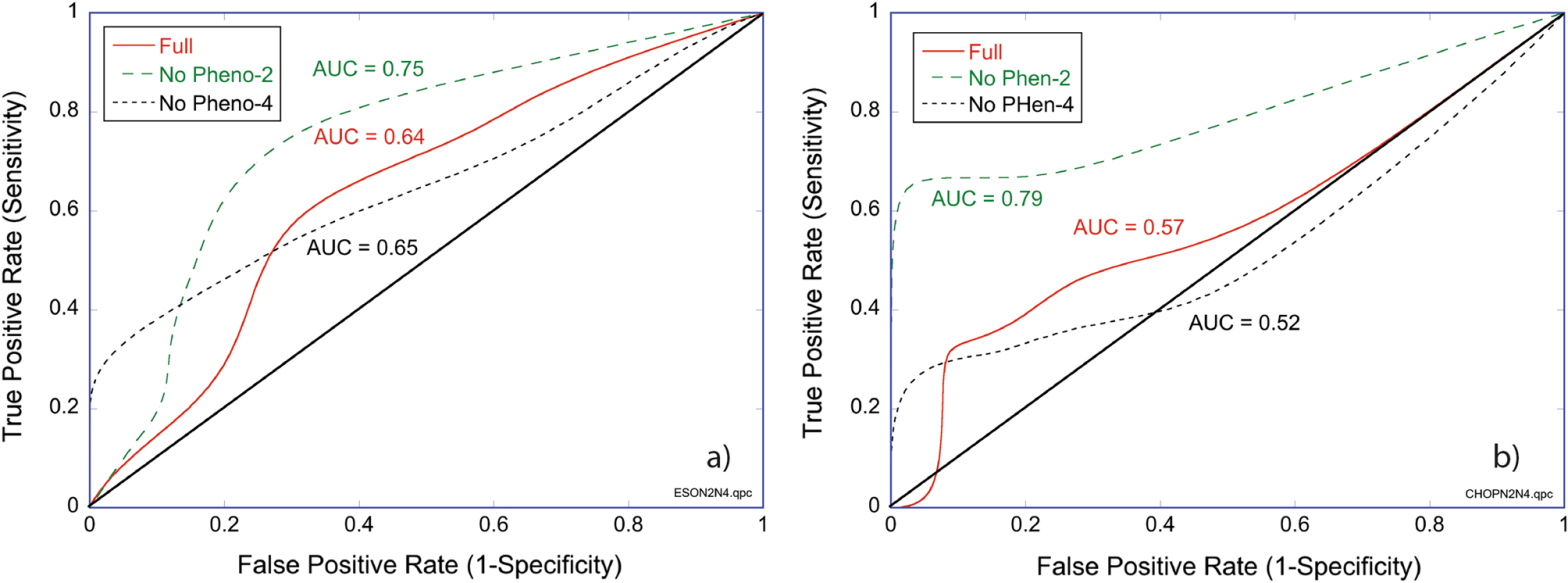
ROC curves when using all wells (Full), when excluding redshifted wells (No Pheno-2), and when excluding mid-frequency enhanced wells (No PHeno-4) during double-hold-out cross-validation of patient predictions. Performance improves when the redshifted wells are removed. (**a**) h-ESO: Full AUC = 0.64, No-Pheno-2 AUC = 0.75, and No-Pheno-4 AUC = 0.65. (**b**) c-BCL: Full AUC = 0.57, No-Pheno-2 AUC = 0.79, and No-Pheno-4 AUC = 0.52.

The relative performances of the predictions are shown as receiver-operator curves (ROC) in Fig. 2a for human esophageal carcinoma and in Fig. 2b for canine lymphoma. The area-under-the-curve (AUC) is given for each case. The three analyses used the full well set (Full), then eliminating phenotype-2 (red-shift) or phenotype-4 (mid-frequency suppression). The striking conclusion from Fig. 2 is the superior performance, based on AUC, of the prediction when the red-shifted wells are eliminated from the analysis, performing markedly better than when using the full set of wells or when only Phenotype-4 was eliminated. An important result of this analysis is the finding, for both human carcinoma and canine lymphoma, that *the red-shifted wells are not representative of the patient response to chemotherapy*. When these wells are censored from the averaging over drug-response replicates, the accuracy of biodynamic profiling improves for predicting patient response to therapy. The explanation of this finding is likely related to the overall health of the individual biopsy samples.

**Figure 3:**
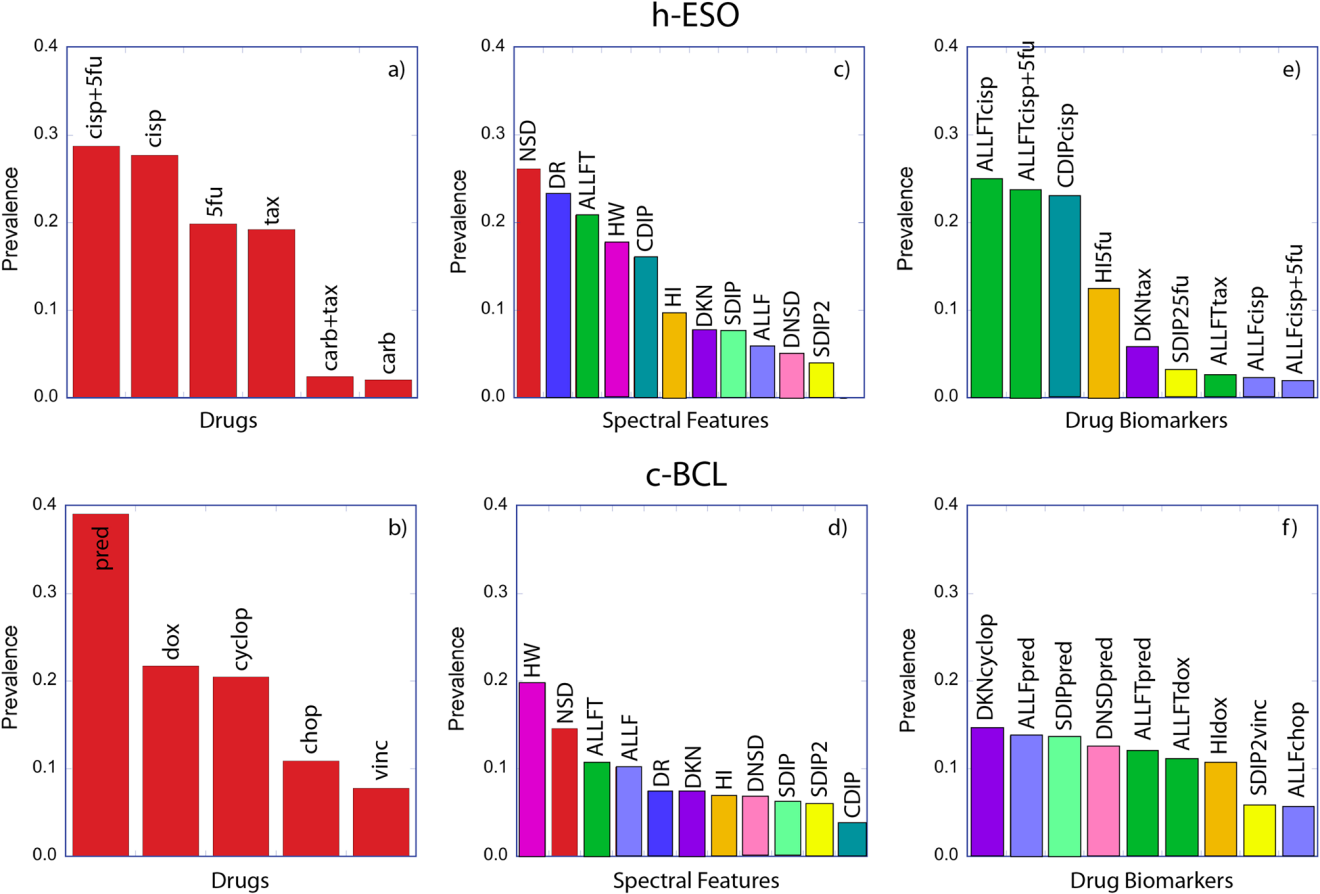
Prevalence of biodynamic features and drugs in the patient prediction for h-ESO and c-BCL. (The features are color-coded for ease of reading.) The preconditions NSD, DR and HW are used in bilinear features as well as linear and are hence highly prevalent. The most important feature is ALLFT which measures broad spectral response with linear time dependence.

During the generation of the ROC curves using the double hold-out cross-validation, the patients are held out prior to the selection of the biomarkers using a within-between (WB) criterion based on the non-held-out training patients for each iteration. Therefore, in addition to building the ROC based on the neural network prediction, the most persistent biomarkers can be identified. These are collectively the most informative as well as the most stable biomarkers that consistently discriminate between the resistant and sensitive cohorts of patients. On each hold-out pass, the biomarkers with the highest WB ratios were identified and tabulated across the 60 hold-out passes. These persistent biomarkers are shown in Fig. 3 for the human esophageal cancer and the canine lymphoma analyses. Figure 3a,b show the drugs which most consistently differentiate between cohorts. For human esophageal patients these are primarily cisplatin and 5-fluorouracil, while for canine lymphoma prednisone is most consistent. Figure 3c,d are the most prevalent features. For both diseases, ALLFT (broad spectral change), NSD (speckle contrast) and HW (average Doppler frequency) are among the most consistent features, representing overall motion suppression increasing with time, average temporal speckle contrast, and the average Doppler frequency, respectively (for a detailed description of feature names and biomarkers, see “Methods” and Supplemental Information Table S5). Figure 3e,f are the feature-drug biomarkers that are most consistent. For human esophageal cancer these are ALLFT associated with cisplatin or cisplatin + 5-fluorouracil as well as CDIP (mid-frequency response) with cisplatin. It is unclear why carboplatin, which has a similar mechanism of action as cisplatin, does not provide strong differentiation in these ex vivo treatments between the sensitive and the resistant cohorts. For the canine lymphoma, there are a number of biomarkers that are roughly equally persistent, including ALLF, ALLFT, DKN (change in average Doppler frequency) and DNSD (change in speckle contrast) associated with cyclophosphamide, prednisone and doxorubicin.

## Discussion

The two major findings in this work are: (1) the heterogeneous well-based biodynamic spectra fall into roughly four phenotypes that are independent of species (human versus canine), disease (carcinoma versus lymphoma) and patient cohort (resistant versus sensitive); and (2) removing the red-shifted wells from the analysis improves the predictive performance of biodynamic profiling for chemosensitivity testing of patients.

The four common well-based spectral phenotypes are a consequence of the general behavior of living tissue irrespective of host species or disease type. Biodynamic imaging measures Doppler frequency shifts of light scattered from cellular and intracellular motions. All living tissue displays common types of cellular motion, including vesicle and organelle transport, membrane motions driven by active cytoskeleton restructuring, and cytoplasmic streaming and cytokinesis. In addition, all living tissue contains actively driven transport such as by molecular motors or cytoskeletal restructuring driven by chemical energy generated by cellular metabolism within the tissues. These universal properties of living tissue translate to common biodynamic spectral phenotypes as observed here.

The existence of universal signatures observed during biodynamic imaging with low-coherence digital holography has important consequences for the eventual application of biodynamic imaging in the selection of cancer therapy. In the previous uses of biodynamic imaging, each different disease and each different species required a full drug-response library to be constructed prior to prediction of patient outcomes. The need to conduct a separate clinical trial for each different indication represented a significant cost of money and time. However, if it can be established that there are universal features or behavior that cross species or disease boundaries, then learning and training from one clinical trial may partially transfer to other diseases, eliminating the need for every aspect of the biodynamic imaging to be repeated for each new disease indication, significantly reducing time and cost required for widespread implementation in clinical oncology. The results of our work presented here are a first step towards showing that such universal trends may be present.

Our second key finding in this work is that, rather than averaging over heterogeneity, it is useful to retain the heterogeneity and identify classes within the well-based spectra. The elimination of well-based phenotypes with low-frequency enhancements (red shifts) prior to the averaging led to improved predictive performance of the biodynamic spectroscopy. Because biodynamic imaging is based on Doppler light scattering, there is an immediate interpretation of the red shift of the non-representative samples. The red shift signifies that the average intracellular activity is decreasing over the duration of the 10-h assays. Lower activity indicates reduced metabolic efficiency that may be interpreted as an overall reduction in sample health, possibly caused by surgical trauma to the sample. Conversely, blue shifts suggest that tissue is increasing its metabolic health through the duration of the assay. In simple terms, this means that the excluded wells with red-shifted samples contained samples whose overall health was already declining prior to drug application. These samples may correspond to areas of a patient tumor where post-biopsy processing caused irreversible loss of ex vivo viability or were ischemic or necrotic at the time of biopsy. For instance, biodynamic metrics have been correlated previously with ATP concentrations measured through ATP assays^34^ which established a direct connection between biodynamic imaging and cellular metabolism. The subsequent censoring, described here, of the red-shifted wells in the analysis of chemosensitivity confirmed that using biodynamic profiles of biopsy response to treatment with a well-based phenotyping approach, retaining heterogeneity rather than averaging over it, improves the accuracy to assess patient chemo-resistance and could improve clinical prediction of drug efficacy in a prospective setting.

## Methods

This study used low-coherence digital holography to perform dynamic-contrast optical coherence tomography (OCT)^20,35,36^ to generate biodynamic profiles of the phenotypic responses of human esophageal cancer and canine B-cell lymphoma to cytotoxic drugs. Tumor heterogeneity was captured in the well-to-well variability of the biodynamic profiles.

### Dynamic contrast OCT with digital holography

**Figure 4:**
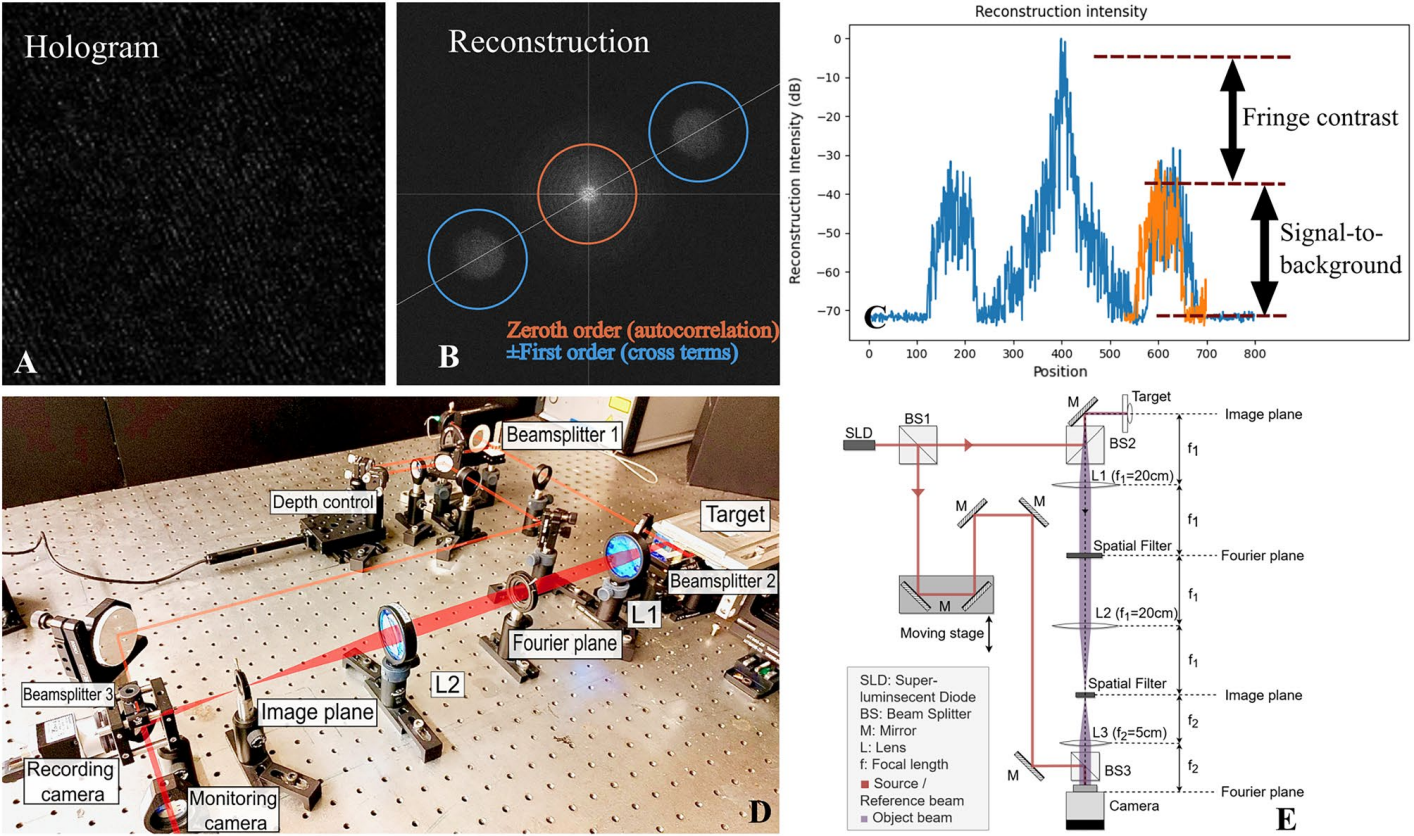
Biodynamic imaging with digital holography. (**A**) Raw hologram zoomed in to show fringes. (**B**) Reconstructed image-domain with phase-conjugate sidebands. (**C**) Sample reflectance through the zero-order and the sidebands (red curve is in the transverse direction). (**D**) Optical configuration with a short-coherence light source and a Mach–Zehnder setup and the hologram captured on the Fourier plane. (**E**) Optical schematic of the layout in (**D**).

Biodynamic imaging (BDI) is a rapid, full-frame, coherence-gated imaging technique that uses short-coherence digital holography to depth-resolve images of millimeter-thick tissue^21^. In BDI, light scattered from a target forms a holographic interference pattern at the Fourier plane by interfering with a plane reference wave in an off-axis spatial heterodyne configuration. The optical system for biodynamic imaging is shown in Fig. 4. The low-coherence light source is a superluminescent diode (SLD) 20.5 mW Superlum Broadlighters Fiber Lightsource (model: S-840-B-I-20 SM) with a center wavelength of 840 nm. The hologram is recorded on a Fourier plane so that image reconstruction is performed using a simple two-dimensional FFT that generates two sidebands. These are phase-conjugate images that represent an optical section of the sample with a section thickness comparable to the coherence length of the light source. Scanning the delay stage shifts the coherence gate through the sample up to 1 mm deep.

Lenses and waveplates condition low-coherence light from the super-luminescent diode before it is split by a polarizing beamsplitter (PBS) into object and reference beams. The object beam is scattered by the target (i.e. tumor biopsy specimen) and then collected and projected onto the Fourier plane (FP) at the camera (CMOS) by a system of lenses with a 3× demagnification to satisfy the conditions of digital holography. The CMOS camera is a Basler camera (Basler acA1920-155um) with a bit depth of 14 bits. Recorded images are 800 by 800 pixels with a square pixel of size of 8 µm. The reference beam passes through the delay stage and is directed onto the camera by means of the mirrors. Lenses condition the reference beam to match the beam profile of the scattered object beam at the camera. The crossing angle is 1.5 degrees, producing a fringe spacing of 32 microns at the operating wavelength of 840 nm. The CMOS pixel array captures the interference pattern with approximately three pixels per holographic fringe and approximately three fringes per speckle transverse correlation length. The hologram is digitally transformed to the image plane through a discrete fast Fourier transform. The digital holography system has a lateral resolution of 20 μm that approximately matches the depth resolution with a field of view of 1 mm.

Images are acquired at 25 frames per second (fps) with an exposure time of 50 ms in stacks of 2000 frames on each pass on each well. The Fourier-domain holograms are numerically converted to the image domain by a two-dimensional spatial FFT. The pixels of the two phase-conjugate sidebands are isolated and converted into time series that are transformed by a 1-dimensional Fourier transform into fluctuation power spectra that are added in quadrature to generate a power spectrum from 10 mHz to 12.5 Hz. Each well is measured on a 40-min period, generating up to 24 “time frames” for each well over the duration of the experiment.

### Drug-response features

The analysis workflow of the fluctuating speckle reconstructed from the speckle holography produces fluctuation spectra for each well of the assay. These are converted into drug-response spectrograms using Eq. (1). In addition to the spectra and spectrograms, there are also measures of the sample that include the temporal speckle contrast, the backscatter brightness of the sample and its reconstructed size. The biodynamic drug response for a specific well of the well plate is converted into a feature vector of 40 feature elements. The details are presented elsewhere^7^ and in the Supplementary Information Figs. S3, S4 and Table S5. As an overview, 9 features are based on global time–frequency decomposition using Legendre polynomials, 9 features are based on local time–frequency decomposition into time and frequency segments, 9 features are based on preconditions of the sample prior to applying the treatment to the well, 9 features are the drug-induced changes of the preconditions, 3 features characterize the baseline prior to drug application, and the final feature is the data quality of the well. Many of these features share strong covariance. Therefore, pooled sets of the 40 features are based on a similarity matrix that pools the similar features into a smaller set of 12 features that are biomarkers of phenotypic drug response. These 12 biomarkers are called ALLF, ALLFT, SDIP, CDIP, HI, SDIP2, DNSD, DKN, NSD, DR, HW, DQ. The general time–frequency behaviors of the biomarkers are given with finer detail in the Supplementary Information Fig. S4 and Table S5 online.

**Figure 5:**
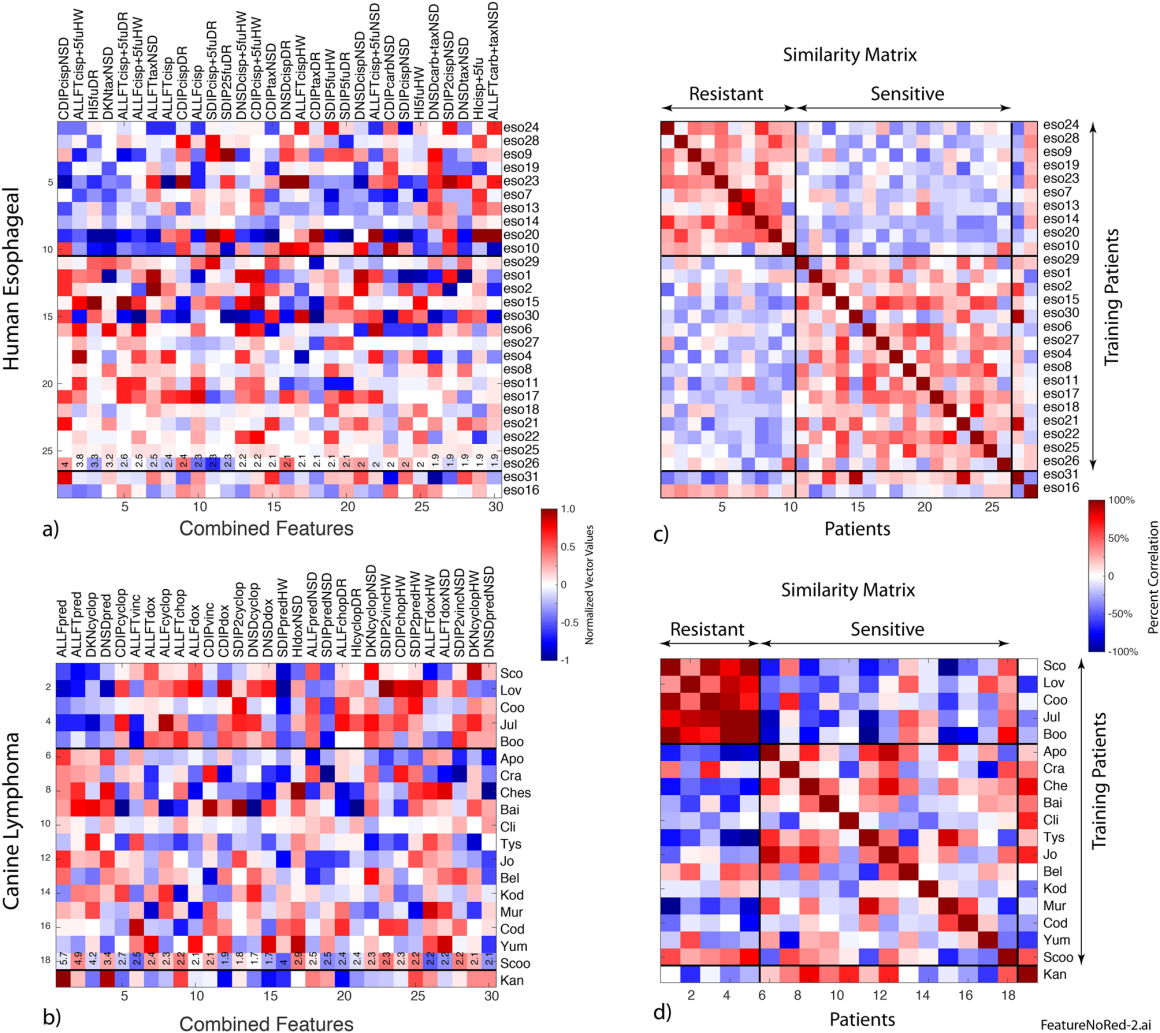
Selected biomarkers (**a**) and (**b**) for each patient in each study including linear and bilinear biomarkers. The similarity matrixes (**c**) and (**d**) are the inner product of the biomarker vectors. The few patients at the bottom were permanently held out of the training because they displayed strong non-representative phenotypes.

Biomarker selection is performed on every iteration of the hold-out cross validation after two patients (one from the resistant cohort and one from the sensitive cohort) are removed from the trial biomarker matrix. This ensures that the selected biomarkers are independent of the hold-out patients. The dominant biomarkers are selected based on the ratio of the within-class standard deviation relative to the between-class standard deviation that sets the WB ratio. Two sets of biomarkers were evaluated: a linear set of the drug-response biomarkers and a bilinear set of the element-wise mean-subtracted product of drug-response biomarkers with the preconditions NSD, DR and HW. The inclusion of the set of bilinear biomarkers captures precondition-dependent biases. For instance, a sample with low NSD may have positive drug response while a sample with high NSD may have negative drug response. The NSD biomarker is a measure of sample metabolic health, with low NSD marking a low metabolism, possibly affecting the drug action. The biomarkers from the linear and bilinear set are combined into the selected biomarkers ranked in decreasing order of their WB ratio and are shown in Fig. 5a for human esophageal and in (b) for canine lymphoma. These selected biomarker vectors are used to construct the similarity matrixes in Fig. 5c,d. In each trial, there were a few patients who had strong non-representative phenotypes and were held out in all the training. For esophageal, these were eso31 who was resistant but displayed a sensitive phenotype, and eso16 who was sensitive but displayed a resistant phenotype. For lymphoma this was Kan who was resistant but displayed a sensitive phenotype. These few nonrepresentative patients are either rare phenotypes for which the trial size is not large enough to establish as a separate class, or they may be patients with a higher degree of tumor heterogeneity that was not fully captured by the number of replicated wells per drug per patient. A larger trial with more replicates could resolve this question in the future.

### Supplementary Information

Supplementary Information 1.

## Data Availability

The data and codes that support the findings of this study are available at Purdue University Research Repository (https://purr.purdue.edu/projects/scirep2023dataandfiles) or from the corresponding author upon reasonable request.
