# Supplementary material for: Comparative oncology chemosensitivity assay for personalized medicine using low-coherence digital holography of dynamic light scattering from cancer biopsies
Source: Sci Rep. 2024 Feb 8;14:2760. doi: 10.1038/s41598-024-52404-w (PMC10853550; doi:10.1038/s41598-024-52404-w)
Supplement: Supplementary file 1 — Supplementary Information 1. [file 41598_2024_52404_MOESM1_ESM.docx]

**Supplemental Information**

**1. Patient Work Flow**

The canine preclinical trial enrolled 19 privately-owned outbred pet dogs who presented with naturally-occurring primary nodal diffuse large B-cell lymphoma (c-BCL) at the Purdue University Small Animal Hospital between August 2015 and December 2016. All methods involving the use of these dogs in this research have been reported in accordance with the ARRIVE guidelines for the reporting of animal experiments (https://arriveguidelines.org).

The study protocol was approved by the Purdue Animal Care and Use Committee (protocol #1307000901) and the Veterinary Clinical Studies Committee (VCSC) at the Purdue College of Veterinary Medicine prior to the enrollment of any dogs. The function of the VCSC is analogous to that of an institutional review board in medical research involving human subjects; it reviews study protocols involving clinical research in client-owned animals to assure that animal welfare is not compromised and that animal owners are appropriately informed of potential research-related hazards. The VCSC approved the informed consent document associated with this study, and the owners of all dogs provided written consent for their dogs to participate prior to enrollment.

All dogs underwent standardized clinical staging tests to determine their eligibility for the study, including complete blood count, serum biochemistry profile, thoracic and abdominal radiography, abdominal ultrasonography, bone marrow aspirate cytology, and electrocardiogram. Dogs were considered eligible for the study if they had a diagnosis of primary nodal DLBCL with favorable performance status (modified Eastern Cooperative Oncology Group status of 0 or 1) and absence of major comorbidities consistent with grade 2 or greater adverse events as defined by the Veterinary Cooperative Oncology Group Common Terminology for Adverse Events (VCOG-CTCAE v1.0) [21]. Dogs were excluded from the study if they had primary extranodal lymphoma or nodal lymphoma of a type other than DLBCL, if they weighed less than 15 kg, if they had high breed-associated risk for carrying the ABCB1-1Δ mutation in the MDR1 gene [22], or if they had received any prior therapy, including glucocorticoids, for their lymphoma.

All dogs underwent surgical lymph node biopsy, and a portion of each dog’s lymph node biopsy was submitted for histopathologic confirmation of disease, while the residual portion was reserved for *ex vivo* BDI. All dogs were treated with 25-week CHOP chemotherapy protocol that consists of alternating, sequential cyclophosphamide, doxorubicin, prednisone, and vincristine, administered as monotherapies on a weekly or biweekly basis. Progression-free survival (PFS) time, defined as the time in days between initiation of chemotherapy and observance of cancer progression or death from any cause, was selected as the primary clinical outcome. Dogs with PFS ≤100 days (placing them in the lowest survivorship quartile for dogs historically treated with CHOP at our institution) were considered non-responders, while those with PFS >200 days were considered responders. The study personnel assessing PFS and those performing biodynamic imaging experiments were blinded with respect to each other’s results until the time of data analysis.

The human trial data comes from patients with a diagnosis of esophageal adenocarcinoma who planned to undergo endoscopic ultrasound (EUS) for staging were seen in the Thoracic Surgery clinic, Medical Oncology clinic, or Gastroenterology clinic at the Indiana University School of Medicine Hospital between April 2015 and January 2020. Patients who appeared to be eligible for this trial based upon performance status and clinical history underwent the Informed Consent Process during the clinic visit and were screened for eligibility prior to their endoscopy procedure. A signed consent and completed eligibility checklist was provided to the Clinical Trials Office prior to registration. All patients were registered with the Indiana University Cancer Center Clinical Trials Office and assigned a unique subject identifier. Regulatory files were maintained by the Clinical Trials Office and applicable regulatory documents were completed and on file prior to registration of any patients. The eligibility criteria for enrolled patients were: 1) Patient with locally advanced esophageal cancer; 2) Patients must be candidates for chemotherapy, radiation and surgical resection; 3) Patients must sign an IRB-approved informed consent. A total of 28 patients were treated with neoadjuvant chemotherapy of either carboplatin+paclitaxel (CT) or cisplatin+5-fluorouracil (CF), selected by clinical preference, followed by re-staging and surgical tumor resection. Patients must be willing to undergo a pinch biopsy at the time of endoscopic ultrasound evaluation performed for staging. Patients with no residual tumor were considered complete responders. Patients with no observable response were considered as non-responders. Patients with either marked or moderate response were considered as partial responders. To create a two-class outcome for the neural network training, the partial responders were assigned to the complete or non-responder classes depending on the class closest in similarity. Further details of the enrollment can be found in Supplementary Note S1 online.

**2. Dog Enrollment for Canine Diffuse B-cell Lymphoma**

The study protocol was approved by the Purdue Animal Care and Use Committee, and written informed consent was obtained from each dog’s owner prior to enrollment. Caliper-based measurement of peripheral lymph nodes was used to determine the objective response to chemotherapy. Progression-free survival (PFS) time, defined as the time in days between initiation of chemotherapy and observance of cancer progression or death from any cause, was selected as the primary clinical outcome. Dogs with PFS ≤100 days (placing them in the lowest survivorship quartile for dogs historically treated with CHOP) were considered non-responders, while those with PFS >200 days were considered responders.

**Table S1. Canine B-cell lymphoma *Ex Vivo* Treatments**

| **Treatment** | **Concentration** |
| --- | --- |
| DMSO | 0.1% |
| 4-hydroxycyclo-phosphamide | 5 μM |
| Doxorubicin | 10 μM |
| Vincristine | 60 nM |
| Prednisolone | 0.6 μM |
| Combination (CHOP) | 5 μM+10 μM  +60 nM+0.6 μM |

**Table S2. Patient Treatments and Outcomes**

| **Dog** | **Treatment** | **Prog. Free Survival (Days)** |
| --- | --- | --- |
| Sco | CHOP | 43 |
| Lov | CHOP | 43 |
| Coo | CHOP | 84 |
| Kan | CHOP | 89 |
| Jul | CHOP | 97 |
| Boo | CHOP | 98 |
| Apo | CHOP | 232 |
| Cra | CHOP | 237 |
| Che | CHOP | 244 |
| Bai | CHOP | 252 |
| Cli | CHOP | 267 |
| Tys | CHOP | 273 |
| Yum | CHOP | 284 |
| Jo | CHOP | 286 |
| Kod | CHOP | 293 |
| Bel | CHOP | 294 |
| Mur | CHOP | 315 |
| Cod | CHOP | 327 |
| Scoo | CHOP | 1018 |

**3. Patient Enrollment for Human Esophageal Adenocarcinoma**

Patients with a diagnosis of esophageal adenocarcinoma who planned to undergo endoscopic ultrasound (EUS) for staging were seen in the Thoracic Surgery clinic, Medical Oncology clinic, or Gastroenterology clinic at the Indiana University School of Medicine Hospital. Of the 28 enrolled patients, 18 received clinical scores based on pathological response in surgical resection specimens, 8 received RECIST scores based on PET imaging, and 2 received no clinical score. At the time of surgical resection, pathological response was defined according to College of American Pathologists guidelines: 1) No residual tumor (complete response, grade 0; 0% tumor); 2) Marked response (grade 1, minimal residual cancer, 0 - <10% residual tumor); 3) Moderate response (grade 2; 10-50% residual tumor); 4) No definite response identified (grade 3, poor or no response; >50% residual tumor).

The patient treatments and outcomes are given in Table S3. All drugs were solubilized in 0.1% dimethyl sulfate (dmso) which is also pipetted into selected wells as a negative control.

**Table S3. Human Esophageal *Ex Vivo* Treatments**

| **Treatment** | **Concentration** |
| --- | --- |
| dmso | 0.1% |
| carboplatin | 25 μM |
| Taxol | 10 μM |
| carboplatin  + Taxol | 25+10 μM |
| cisplatin | 25 μM |
| 5FU | 25 μM |
| cisplatin  + 5FU | 25+25 μM |

**Table S4. Patient Treatment^#^ and Outcomes***

| **Patient** | **Treatment** | **Clinical Outcome** |
| --- | --- | --- |
| Eso1 | carbo+tax | SD, no path. |
| Eso2 | carbo+tax | PR, minimal path. |
| Eso9 | carbo+tax | SD, no path. |
| Eso16 | carbo+tax | SD, no path. |
| Eso7 | carbo+tax | PR (mod) |
| Eso23 | carbo+tax | SD, no path. |
| Eso24 | carbo+tax | SD, no path. |
| Eso28 | carbo+tax | SD, no path. |
| Eso29 | cisp+5fu | SD, no path. |
| Eso31 | carbo+tax | SD, no path. |
| Eso13 | oxaliplatin+5fu | PR (mod) |
| Eso14 | cisp+5fu | PR (mod) |
| Eso15 | carbo+tax | PR, no path. |
| Eso20 | carbo+tax | PR |
| Eso30 | cisp+5fu | PR (marked) |
| Eso4 | cisp+5fu | PR (marked) |
| Eso6 | carbo+tax | PR (marked) |
| Eso27 | cisp+5fu | PR (marked) |
| Eso8 | cisp+5fu | CR |
| Eso10 | carbo+tax | CR |
| Eso11 | cisp+5fu | CR |
| Eso17 | cisp+5fu | CR |
| Eso18 | carbo+tax | CR |
| Eso21 | carbo+tax | CR |
| Eso22 | carbo+tax | CR |
| Eso25 | carbo+tax | CR |
| Eso26 | carbo+tax | CR |
| Eso19 | FF | CR |

# All patients received radiation therapy in addition to the chemotherapy.

* If no pathology, then RECIST is used.

**4. Data Structure and Data Flow for Machine Learning**

To analyze the correspondence between drug-response spectrograms and patient outcomes, a workflow was established starting upstream with the time-stack of holographic reconstructions of the individual wells. Frames were acquired at 25 fps for up to 2000 frames. Earlier data formats used two acquisitions, one at 25 fps for fast processes and the other at 5 fps for slow processes, and the spectra were stitched. The stack of holograms was transformed into the image domain by a 2D spatial fast Fourier transform. One of the sidebands was isolated as the optical section of the sample, a binary mask was generated, multiplied by the reconstructions, and the stack of masked reconstructions was sent through a fast Fourier transform along the time axis. Normalization of the Fourier transform was based on Parseval’s theorem to compensate the effect of fringe washout during holographic recording. The modulus squared of the normalized Fourier transform was summed for each pixel into a power spectrum for each well at each time loop. The wells were remeasured every 40 minutes for the duration of the assay. At this stage, each well is characterized by a power spectrum at 40-minute intervals. The spectrogram for each well is generated by taking the difference in log spectral density using an average over the pre-treatment baseline as the reference, where

$$D\left(\omega,t\right)=\log S\left(\omega,t\right)-\log S\left(\omega,t_{0}\right)$$

is the differential drug-response spectrogram. For small relative changes, this is equal to the fractional change in spectral content, whereas for large changes, the differential drug-response spectrogram grows logarithmically which helps limit outlier behavior. Extreme outlier behavior, for instance caused by a small shift in the sample position during pipetting, is further reduced by using a hyperbolic tangent with a maximum allowable deviation (MAD) limit set to 1.5 to limit the spectrogram shifts. In addition, each spectrogram is assigned a data quality metric DQ based on signal strength and stability. Averaged spectrograms are weighted averages using the DQ as the weight factor. For wells that are extreme outliers, typically the DQ is set to a veto value of zero.

***Spectrogram Heterogeneity***

Tissue dynamics spectroscopy (TDS) of a living biopsy sample measures the relative change in the Doppler fluctuation spectrum in response to an applied therapeutic or other perturbation of the biopsy. A single biopsy of approximately 50 mm^3^ is separated using a scalpel into approximately 32 samples of 1 mm^3^ volume each that are placed into a multiwell plate. Several of these samples receive replicate treatments. However, the TDS drug spectrograms across these subsets of a biopsy may display several different phenotypes related to the baseline conditions. While previous work identified and characterized the average spectral response of the samples, most samples have a more complicated drug response structure. Therefore, in this work, the full well-to-well variability is retained for downstream analysis.

The inter-sample variability in the TDS signatures, originating from tumor heterogeneity, poses a challenge for the prediction of patient response to therapy. The drug-response spectrograms from a single patient (Esophageal patient 31) are shown in Fig. S1 as an example of the heterogeneity of the multiple spectrograms. The horizontal axis shows frequencies in the range from 10 mHz to 10 Hz. The vertical axis stands for drug response time. The solid blue line is when the treatment was applied. The axes are the same for all spectrograms. This patient was treated with DMSO (0.1%), Carboplatin (25µM), Taxol (10µM), 5FU fluorouracil (25µM), Cisplatin (25µM) and the combo of Carboplatin and Taxol (25µM+10µM).

Figure S1 (a) shows the averaged drug-response spectrograms, and Figure S1 (b) shows the spectrogram collage for each well. The replicate number for each treatment is three or four. As an example of the well-to-well variability, the Carboplatin drug-response spectrograms under the same treatment show different spectrograms for each well. The first well shows a low-frequency suppression and a slight mid-frequency enhancement (less than 10% change of power density). The second and the third wells show a relatively stronger low-frequency and high-frequency suppression, and a mid-frequency enhancement. The fourth well shows a strong low-frequency enhancement and a deep mid -and high-frequency suppression. The wells for Taxol show similar variability. The heterogeneity of these spectrograms makes averaged spectrograms of the replicate wells a compromise that neutralizes the different well-based phenotypes that contribute nonlinear information to the training of the neural network.


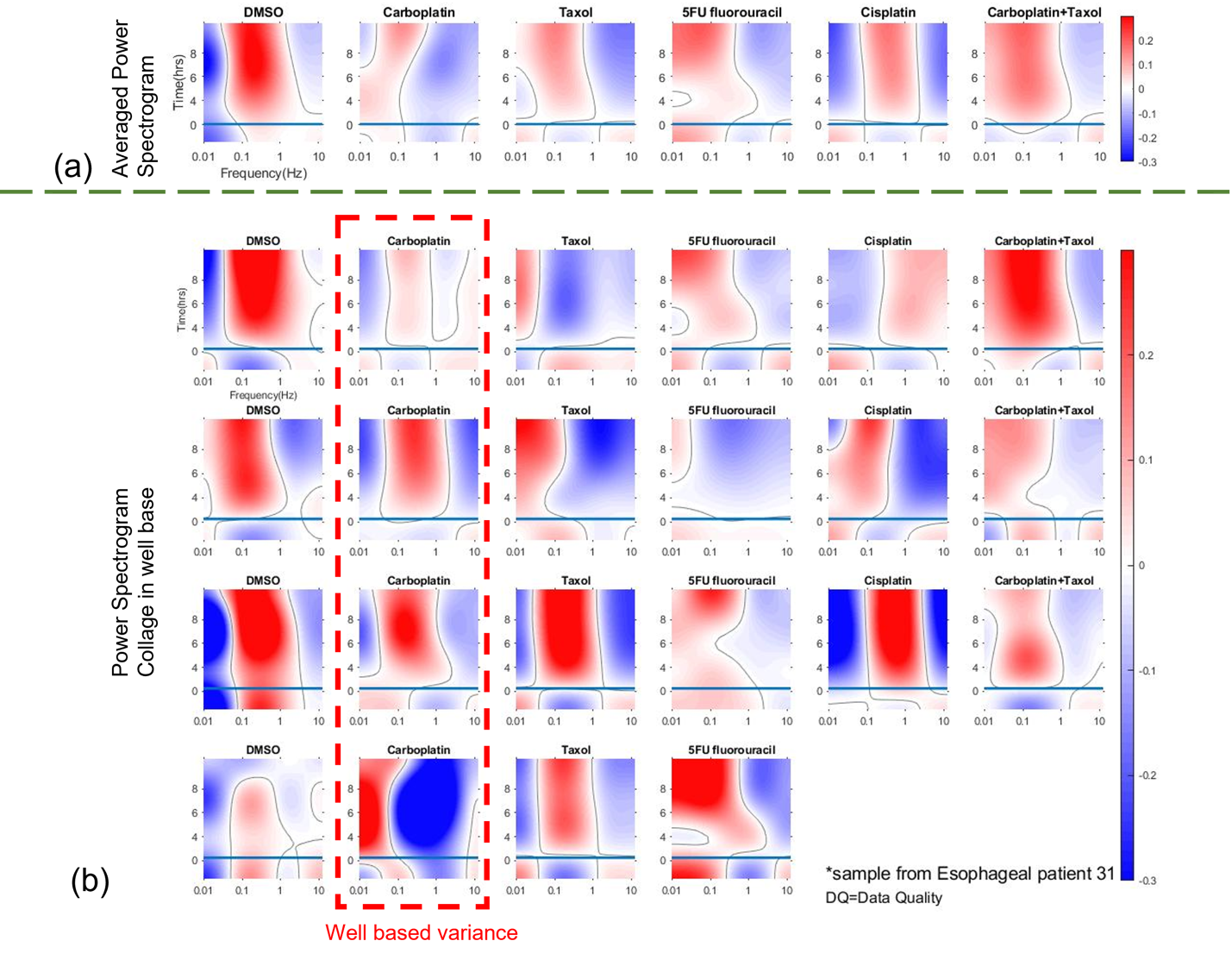


Fig. S1 Averaged drug-response spectrograms from a single patient of biopsy sub-samples in individual wells treated with DMSO (0.1%), Carboplatin (25µM), Taxol (10µM), 5FU fluorouracil (25µM), Cisplatin (25µM) and the combination therapies of Carboplatin and Taxol and Cisplatin and 5FU. The horizontal axis shows frequencies in the range from 10 mHz to 10 Hz. The vertical axis is the experiment duration. The solid blue line is when the treatments were applied. The axes are the same for all spectrograms. (a) The averaged drug-response spectrograms from Esophageal patient 31. (b) The spectrogram collage from the same patient for individual wells. The replicate number for each treatment is three or four.

**5. Average Spectrograms with Red-Shifted Wells Censored**

The averaged drug-response spectrograms with the red-shifted wells removed are shown in Fig. S2a) for the human esophageal resistant and sensitive cohorts and in Fig. S2b) for the canine lymphoma resistant and sensitive cohorts. The average differences between resistant and sensitive spectrograms are shown in the respective bottom rows. The drug-response spectrograms are DMSO-subtracted, which means that the average negative control is subtracted from the drug-response spectrograms. The biopsy samples are living and relatively healthy, but there is drift in the sample metabolism, either through recovery from the trauma of surgery, or from slow responses to the laboratory environment (the samples are temperature-controlled to physiological temperature, but there is no control of CO_2_ during the experiment). The raw red-shifts were already censored in the averages, but the drug-response spectrograms in Fig. S2 can still show a relative red shift compared to the average DMSO response, for instance, carboplatin and taxol in the resistant groups of esophageal patients are red-shifted relative to the DMSO response.

For both human and canine, and for both carcinoma and lymphoma, there is a striking difference in the DMSO-subtracted drug-response spectrograms between the resistant and the sensitive patient cohorts. These differences are further emphasized in the bottom row showing the difference of resistant relative to sensitive spectrograms. For the case of h-ESO, many of the drug responses of the resistant cohort are red-shifted relative to the sensitive cohort. On the other hand, this trend is not seen for c-BCL, where the doxorubicin treatment displays a blue shift and the prednisone response shows overall inhibition of activity. An interesting comparison is the behavior of the carrier DMSO which is taken as the negative control. The DMSO response was averaged separately for each cohort (resistant versus sensitive) and the difference of the spectral responses are shown at the bottom row. For both h-ESO and c-BCL, the resistant DMSO is red-shifted relative to the sensitive cohort. The absolutely red-shifted wells have already been censored from these data, but there can still be relative red shifts. This suggests that the general sample response of the resistant cohort, even when untreated, tends to lower activity relative to the sensitive cohort samples, possibly because of different metabolic demands of the cancer cells comprising the tissues of the resistant relative to the sensitive cohorts.

Fig. S2 Averaged drug-response spectrograms after removal of red-shifted wells. The horizontal axis is frequency from 10 mHz to 10 Hz. The vertical axis is time, extending 4 hours before treatment and up to 12 hours after treatment. The top rows are the resistant (R) cohort, and the middle rows are the sensitive (S) cohort. The bottom rows are the difference of R – S. The replicate numbers are on the lower left of each spectrogram.

**6. Features and Pooled Biomarkers**

The biodynamic response of a given well containing a sub-sample of the biopsy is converted to a 40-element vector. Each element is a “feature”. The 40 features represent the pre-condition of the sample prior to in vitro treatment (8), the change in the condition (8) by the end of the experimental period (approximately 16 hours), time-frequency features (18), baseline properties (3) and well data quality (1). The pre-condition features, the change and the baseline and quality features are explained in Fig. S3. The time-frequency features are best understood through their spectral filters, also shown in Fig. S3. There are 9 “global” filters and 9 “local” filters. The global filters are Legendre polynomials in time and frequency. ALLF = “all frequencies”, SDIP = “sine-dipole”, CDIP = “cosine-dipole”. The numerical suffix is the 0^th^, 1^st^ and 2^nd^ Legendre polynomial. The local features are also understood through their time-frequency filters. LOF = “low frequency”. MID = “mid-frequency”, and HI = “high-frequency”, with the same Legendre suffix.

Fig. S3 The time-frequency filters for the global and local spectral drug response features of which there are 18. The preconditions relate to the properties of the sample prior to the application of in vitro treatment, of which there are 8. There are a corresponding 8 that measure the change in those conditions between the end and start of the experiment. There are 3 baseline features that capture frequency shifts of the baseline prior to treatment. The last is a data quality metric that reflects sample size, health and stability.

A central step in the analysis is the pooling of individual features to benefit from signal averaging. Clustered correlation matrices of the individual features are shown in Fig. S4 for ESO and BCL. Most features have correlations with other features, with many similarities between h-ESO and c-BCL. These correlations (shown in the clustered similarity matrices in Fig. S4) are used for unsupervised hierarchical clustering. The clustering dendrograms were “pruned” to “pool” several original features into a new pooled feature. The pooling selection is shown at the bottom in Fig. S4. For instance, the pooled feature “ALLF” is the linear combination of ALLF0, MID0, -ALLF2 and -MID2. The minus signs indicate that that feature was anti-correlated with the others. The pooled feature name is taken from the dominant behavior of the group of original features. For instance, ALLF captures broad-frequency response to the drug. The same pooling was used for both h-ESO and c-BCL. The physiological interpretations of the resulting 12 pooled features are explained in Table S5.

Fig. S4 Clustered correlation matrices for h-ESO and c-BCL features. Many of the correlations are shared between the species (human and canine) and the types of cancer (esophageal and lymphoma). Individual features (global, local, preconditions, change in preconditions, and baseline and quality) that are pooled into 12 biomarkers that were used for the chemosensitivity analysis.

**Table S5 Pooled Biomarkers**

| **Pooled Feature** | **Description** | **Pooled Feature** | **Description** |
| --- | --- | --- | --- |
| ALLF | Broadband frequency response independent of time | DNSD | Change in temporal speckle contrast |
| ALLFT | Broadband frequency response with linear time dependence | DKN | Change in the knee frequency |
| SDIP | Blue shift (positive) versus red shift (negative) | NSD | Temporal speckle contrast |
| CDIP | Mid-frequency enhancement (positive) versus suppression (negative) | DR | Dynamic range of low relative to high frequency spectral density |
| HI | High-frequency enhancements | HW | Knee frequency |
| SDIP2 | Non-monotonic drug response | DQ | Data quality |

***Bilinear Biomarkers***

A linear drug-response biomarker is a pair of a drug and a biomarker. For instance, ALLFpred is the value of the ALLF pooled feature for the drug prednisone applied to a canine lymphoma biopsy. Example of these drug-response biomarkers are shown in Fig. 5(a) and (b) of the text.

In addition, a bilinear drug-response biomarker is the product of a linear biomarker value with one of the pooled precondition features NSD, HW or DR. For instance the drug-response biomarker CDIPcispNSD is the product of CDIPcisp with NSD for that well. These bilinear combinations capture conditional dependences. For instance, if the health of a sample is low, leading to small values of NSD, this can relate to an increased value for a linear biomarker, and vice versa. This use of products of biomarkers is well established in machine learning as part of the kernel approach for classification applications. In Fig. 5 of the text, both linear and bilinear biomarkers are selected after a pair of patients (one sensitive and one resistant) are randomly selected and held out of the feature vector matrix, and this process is repeated 60 times as the selected biomarkers are used in a down-stream neural network classifier. The values shown in Fig. 5 are for just one of these iterations.

**7. Neural Network Classifier**

A neural network classifier was developed for this project to perform dimensionality reduction, taking 30 features down to 3 latent values. The neural network structure consisted of a triplet loss function operating on a network architecture that had 30 input neurons, two hidden fully-connected layers of only 8 neurons each, and an output layer of 3 neurons. The triplet loss algorithm [1] selects an anchor data vector from the feature vector matrix, then selects a data vector from the same training class plus a data vector from outside the training class. This triplet of data vectors is fed through the feed-forward neural network to generate a three-dimensional output. The objective function minimizes the distance in latent space between the similar data and the anchor data, while maximizing the distance between the dissimilar data and the anchor data. Successive training acts to cluster similar inputs together and to separate clusters of dissimilar inputs.

The neural networks were built “by hand” from the Matlab Deep Learning Toolbox. Training used minibatches of 180, validation fraction of 20%, learning rate of 1x10^-4^, gradient decay of 0.9 and gradient decay squared of 0.99. The network was trained for 750 epochs while monitoring the decrease of the loss function evaluated on the 20% validation set. After dimensionality reduction, the three-dimensional latent space is used in a max-margin classifier to classify the hold-out patients.

**8. Data Availability**

The data and codes that support the findings of this study are available at GitHub from https://github.rcac.purdue.edu/nolte /SciRep2023 -Data-and-Files or from the corresponding author upon reasonable request.

1. Kaya, M. and H.S. Bilge, *Deep Metric Learning: A Survey.* Symmetry-Basel, 2019. **11**(9).
